# Generative artificial intelligence in graduate medical education

**DOI:** 10.3389/fmed.2024.1525604

**Published:** 2025-01-10

**Authors:** Ravi Janumpally, Suparna Nanua, Andy Ngo, Kenneth Youens

**Affiliations:** Clinical Informatics Fellowship Program, Baylor Scott & White Health, Round Rock, TX, United States

**Keywords:** Generative AI, LLM, gpt, GME, graduate medical education, ChatGPT, artificial intelligence, education

## Abstract

Generative artificial intelligence (GenAI) is rapidly transforming various sectors, including healthcare and education. This paper explores the potential opportunities and risks of GenAI in graduate medical education (GME). We review the existing literature and provide commentary on how GenAI could impact GME, including five key areas of opportunity: electronic health record (EHR) workload reduction, clinical simulation, individualized education, research and analytics support, and clinical decision support. We then discuss significant risks, including inaccuracy and overreliance on AI-generated content, challenges to authenticity and academic integrity, potential biases in AI outputs, and privacy concerns. As GenAI technology matures, it will likely come to have an important role in the future of GME, but its integration should be guided by a thorough understanding of both its benefits and limitations.

## 1 Introduction

Generative artificial intelligence (GenAI) is a relatively new technology that uses advanced machine learning models to generate humanlike expression. Large language models (LLMs) like ChatGPT (OpenAI, San Francisco, United States) rely on a machine learning architecture called a “transformer.” A key feature of transformers is their self-attention mechanism, which allows the model to assess the importance of words in a sequence relative to one another, enhancing its ability to understand context and, when trained on vast amounts of data, resulting in a remarkable ability to understand and generate humanlike text (1). Such models excel at tasks like document summarization, sentiment analysis, question answering, text classification, translation, text generation, and as conversational chatbots. Related models called large vision models (LVMs), Vision-Language Models (VLMs), large multimodal models (LMMs), diffusion models, and generative adversarial networks (GANs) provide similar or overlapping functionality for image, audio, and video processing and generation. It is widely believed that GenAI will have far-reaching societal impact and will be incorporated into multiple aspects of our daily lives (2, 3). GenAI has the potential to revolutionize multiple industries, with healthcare and education among the likely targets.

In healthcare, GenAI has shown promise in a broad range of applications such as clinical decision support, medical education, clinical documentation, research support, and as a communication tool (4). GenAI models like ChatGPT, even without special fine-tuning for medical knowledge, achieve performance at or near the passing threshold on all three United States Medical Licensing Examination (USMLE) Step exams (5). Studies evaluating performance on medical specialty board examination-or in-service examination-level questions have shown mixed results, but in some cases LLM performance has approached that of senior medical trainees (6–9). GenAI-powered tools are deployed in production clinical environments today, most notably in the patient care-adjacent domains of clinical documentation (10) and provider-patient communication, where they have shown promise in improving EHR-related provider inefficiency and burnout (11, 12).

In the medical educational setting, GenAI potentially offers multiple benefits such as easy personalization of learning experiences, simulation of real-world scenarios and patient interactions, and practicing communication skills (13). These potential gains are balanced by meaningful risks, such as the trustworthiness of AI-generated content, the deepening of socioeconomic inequalities, and challenges to academic integrity (14, 15).

Graduate medical education (GME) shares many characteristics with undergraduate medical education and with other types of healthcare education. As adult learners, medical trainees are theorized to learn best when self-motivated, self-directed, and engaged with task-centered, practical topics (16). Historically, medical education used time spent in the training environment as a proxy for learning success. More recently, there has been renewed interest in competency-based medical education (CBME), a paradigm that uses achievement of specific competencies rather that time spent (or other structural measures) as the key measure of learning success (17, 18). CBME serves as the foundation of the Accreditation Council for Graduate Medical Education (ACGME)’s accreditation model, and is the key theory underpinning the formative “Milestones” used by ACGME-accredited programs to assess trainee development and to improve education (19).

Having built a foundation in medical sciences and basic clinical skills in medical school, GME trainees spend little time in the classroom, with most of their learning occurring with real patients as they function as members of the healthcare team. A core tenet of GME is “graded authority and responsibility,” where trainees progressively gain autonomy until they achieve the skills to practice independently. Additionally, trainees are expected to become “physician scholars”; participants in ACGME-accredited GME programs participate in scholarly pursuits like research, academic writing, quality improvement, and creation of educational curricula (20).

In this paper, we present concise summary of the existing literature (Table 1) and commentary on the potential opportunities and risks of GenAI in the GME setting.

**Table 1:** Literature on GenAI in the GME setting.

Specialty	First author (Publication date)	Title (Citation)	Brief description
Administration	Mangold, S (2024)	Artificial Intelligence in Graduate Medical Education Applications (101)	Commentary on the use of GenAI in GME application materials.
Administration	Quinonez, S (2024)	ChatGPT and Artificial Intelligence in Graduate Medical Education Program Applications	Commentary on the use of GenAI in GME application materials.
Administration	Zumsteg, J (2023)	Will ChatGPT Match to Your Program? (97)	Commentary on the use of GenAI in GME application materials.
Anesthesiology	Sardesai, N (2023)	Utilizing Generative Conversational Artificial Intelligence to Create Simulated Patient Encounters: A Pilot Study for Anaesthesia Training (48)	Study using an LLM to simulate patient conversations for trainees regarding certain anesthesia procedures. The tool showed good accuracy in simulating patient responses and behavior.
Dermatology	Ayub, I (2023)	Exploring the Potential and Limitations of Chat Generative Pre-trained Transformer (ChatGPT) in Generating Board-Style Dermatology Questions: A Qualitative Analysis	Study using an LLM to generate board exam-style dermatology questions, showing poor performance of the model in generating accurate and appropriate questions.
Dermatology	Breslavets, M (2024)	Advancing dermatology education with AI-generated images.	Commentary with examples using a GAN to generate synthetic clinical images for dermatology training.
Dermatology	Lim, S (2024)	Exploring the Potential of DALL-E 2 in Pediatric Dermatology: A Critical Analysis	Study using a diffusion model to generate synthetic clinical images of dermatologic conditions for dermatology training, showing poor performance of the model for most tested conditions.
Emergency Medicine	Barak-Corren, Y (2024)	Harnessing the Power of Generative AI for Clinical Summaries: Perspectives From Emergency Physicians (32)	Study using an LLM to generate clinical supervisory notes, showing a significant reduction the in time and effort required to create notes, without any reduction in note quality on simpler notes.
Emergency Medicine	Webb, J (2023)	Proof of Concept: Using ChatGPT to Teach Emergency Physicians How to Break Bad News (49)	Proof-of-concept study using ChatGPT to roleplay breaking bad news to patients.
Neurosurgery	Arfaie, S (2024)	ChatGPT and Neurosurgical Education: A Crossroads of Innovation and Opportunity (90)	Review of the literature and summary of the uses of GenAI for educating neurosurgical trainees.
Neurosurgery	Bartoli, A (2024)	Probing Artificial Intelligence in Neurosurgical Training: ChatGPT Takes a Neurosurgical Resident’s Written Exam (111)	Study evaluating the performance of an LLM in generating board examination-style questions, showing poor performance of the LLM in generating a small trial set of exam-quality questions.
Neurosurgery	McLean, A (2024)	Application of Transformer Architectures in Generative Video Modeling for Neurosurgical Education (112)	Detailed description of a planned study that would use a diffusion model to create synthetic neurosurgical training videos.
Ophthalmology	Sevgi, M (2024)	Medical Education with Large Language Models in Ophthalmology: Custom Instructions and Enhanced Retrieval Capabilities (113)	Description of tools using LLMs to teach clinical guidelines in ophthalmology and to summarize current ophthalmology research.
Orthopedic Surgery	DeCook, R (2024)	AI-Generated Graduate Medical Education Content for Total Joint Arthroplasty: Comparing ChatGPT Against Orthopedic Fellows (114)	Study using an LLM to generate educational summaries of total joint arthroplasty-related topics, showing that the LLM created better orthopedic training content than orthopedic fellows across several topics and domains.
Pathology	Cecchini, M (2024)	Harnessing the Power of Generative Artificial Intelligence in Pathology Education (18)	Review of the literature and summary of the uses of GenAI for educating pathology trainees.
Pediatrics	Ba, H (2024)	Enhancing Clinical Skills in Pediatric Trainees: A Comparative Study of ChatGPT-Assisted and Traditional Teaching Methods (115)	Study comparing LLM-assisted instruction with traditional instruction on pediatric clinical skill education, showing comparable or better performance of the LLM-assisted method.
Pediatrics	Suresh, S (2024)	Large Language Models in Pediatric Education: Current Uses and Future Potential (116)	Review of the literature and summary of the uses of GenAI for educating pediatrics trainees, showing that LLM-assisted instruction did not affect theoretical knowledge application but did enhance practical clinical skills.
Pediatrics	Waikel, R (2023)	Generative Methods for Pediatric Genetics Education (56)	Study using synthetic images of individuals with uncommon genetic conditions to train pediatric residents, showing that the synthetic images performed similarly but were slightly less helpful than real patient images.
Primary Care	Parente, D (2024)	Generative Artificial Intelligence and Large Language Models in Primary Care Medical Education (59)	Review of the literature and summary of the uses of GenAI for educating primary care trainees.
Radiology	Lyo, S (2024)	From Revisions to Insights: Converting Radiology Report Revisions into Actionable Educational Feedback Using Generative AI Models (60)	Study using an LLM to compare preliminary (trainee) and finalized radiology reports, identify discrepancies, and suggest review topics. The LLM consistently and accurately identified discrepancies and suggested relevant feedback.
Radiology	Meşe, I (2024)	Educating the Next Generation of Radiologists: A Comparative Report of ChatGPT and E-Learning Resources (117)	Review of the literature and summary of the uses of GenAI for educating radiology trainees.
Radiology	Mistry, N (2024)	Large Language Models as Tools to Generate Radiology Board-Style Multiple-Choice Questions (61)	Study using two LLMs to generate board exam-style radiology questions, demonstrating that one LLM generated questions of equivalent quality to real American College of Radiology in-service exam questions.
Surgery	Lia, H (2024)	Cross-Industry Thematic Analysis of Generative AI Best Practices: Applications and Implications for Surgical Education and Training (118)	Analysis of ethical considerations when integrating GenAI into surgical education, with example use cases.
Surgery	Sathe, T (2024)	How I GPT It: Development of Custom Artificial Intelligence (AI) Chatbots for Surgical Education (119)	Commentary on the use of GenAI chatbots for surgical education with description of several potential use cases.

Summary of existing literature of which we are aware focusing on GenAI in GME. The summary excludes papers focused on mainly on testing LLM performance on medical knowledge tasks, papers on non-GME-specific clinical, educational or academic applications of GenAI, and papers about artificial intelligence in general.

## 2 Opportunities

### 2.1 EHR workload reduction

Given their long work hours and stressful work environment, GME trainees are particularly susceptible to burnout, with rates higher than their age-matched peers in non-medical careers and higher than early-career attending physicians (21). Burnout among the academic physicians who comprise most GME faculty also occurs, and may impact the quality of training they are able to deliver (22, 23). Thus, innovations that prevent overwork and burnout have the potential to benefit GME trainees and faculty.

One unintended consequence of the adoption of electronic health records (EHRs) has been a dramatic increase in time spent in documenting clinical encounters. Many physicians now spend as much time documenting in the EHR as they do in patient-facing activities (24). This documentation burden can result in medical errors, threats to patient safety, poor quality documentation, and attrition, and is a major cause of physician burnout (25). Various strategies have been tried to reduce physician documentation burden, including medical scribes and various educational interventions, workflow improvements, and other strategies (26). Given its ability to summarize, translate and generate text, GenAI demonstrates clear potential as a technological aid to alleviate the burden of clinical documentation. The most notable current application is ambient listening tools that use GenAI to transcribe and analyze patient-doctor conversations, converting them into structured draft clinical notes that the physician would theoretically only then need to review for accuracy. Numerous organizations are piloting such technology as of the time of this writing (27), though the few results published so far about real-world performance have been mixed (10, 28, 29). Examples of other less commercially mature concepts for how GenAI could reduce clinical documentation burden include tools to improve medical coding accuracy (30), to generate clinical summary documentation like discharge summaries (31), and to draft GME faculty supervisory notes (32).

In addition to documenting clinical encounters, physicians (including GME trainees) spend large amounts of time in the EHR managing inbox messages, including patient messages, information about tests results, requests for refills, requests to sign clinical orders, and various administrative messages (33). As another major contributor to workload, EHR inbox management is also a cause of burnout (34, 35). This problem came to be of particular importance during the COVID-19 pandemic, where patient messaging increased by 157% compared to pre-pandemic levels (35). LLMs have shown the ability to draft high-quality, “empathetic” responses to patient questions (36). Early efforts to use LLMs for drafting replies to patient inbox messages have shown promising results, with multiple studies showing that LLMs can draft responses of good quality (37, 38) and at least one study showing good provider adoption with significant reductions in provider assessment of multiple burnout-related metrics (11). Multiple health information technology companies, including the largest United States EHR vendor, have already brought GenAI functionality for EHR inbox management to market (39–41).

### 2.2 Clinical simulation

Simulation-based medical education (SBME) has evolved significantly since the early use of mannequins for basic life support training 60 years ago, and simulation using high-fidelity mannequins and virtual and augmented reality tools are now a vital component of GME. There is a substantial body of evidence confirming the benefits of simulation-based training and the successful transfer of these skills to real patients (42, 43). Simulations are used both to educate and to assess performance in GME. For example, the American Board of Anesthesiology incorporates an Objective Structured Clinical Examination (OSCE) meant to assess communication and professionalism, as well as technical skills, into the board examination process for anesthesiology residents (44). Many of the current applications of SBME in GME are targeted at procedural skills like complex surgical techniques, bridging the gap for trainees’ experiential learning on invasive, uncommon, or high-acuity procedures (45). The integration of artificial intelligence into clinical simulations would theoretically allow for the customization of scenarios based on a trainee’s skill level and performance data, providing a personalized learning experience and potentially opening the door to new types of patient simulation (43). Accordingly, there has been interest in using conversational GenAI to simulate patient encounters to practice cognitive and communication skills, though this application is more often focused on undergraduate medical education (15, 33, 46–49).

Among the most interesting potential applications of GenAI in GME is the concept of using synthetic data as training material for visual diagnosis. GANs and diffusion models have shown promise in generating realistic images of pathology findings (50, 51), skin lesions (52–54), chest X-rays (55), genetic syndromes (56), and ophthalmological conditions (57). The synthetic data approach may ultimately address important limitations in image-based training data sets, such as underrepresentation of certain patient demographics and adequate demonstration of rare findings.

### 2.3 Individual education

Individualized tutoring produces better academic outcomes than learning in a traditional classroom setting (58). Skilled teachers can guide learners at different levels through complex topics, offering tailored and accessible explanations. One-on-one tutoring delivered by humans is costly, and skilled teachers are not available everywhere, but GenAI tools may have some of the same benefits at a fraction of the cost. LLMs show promise as a tool for explaining challenging concepts to graduate medical trainees in a manner tailored to the learner’s level (18), and LLMs could be configured to act as personalized tutors (59). In one study, an LLM successfully reviewed trainee-generated radiology reports and generated relevant educational feedback, a concept which could be extended to other types of clinical documentation (60).

GME trainees preparing for board examinations often use question banks to study, and GME programs use board-exam style questions to assess trainee progress. Question generation can be a costly and labor-intensive proposition (61). Authors report mixed success with using LLMs to generate board exam-style questions (61, 62), but as the technology matures, it seems likely that LLMs will be used by trainees and educators alike to create high-quality self-directed study materials and test questions.

### 2.4 Research and analytics support

LLMs are powerful tools for academic research and writing, and can assist in idea generation, processing complex background information, and proposing testable hypotheses (63, 64). LLMs readily summarize complex academic papers and draft academic text, abilities that can accelerate academic productivity (65). When paired with reliable academic databases and search engines and/or when fine-tuned with specific knowledge, LLMs do a serviceable job of conducting literature reviews (66, 67), synthesizing findings from existing literature, and drafting new scientific text with accurate literature citations (68). LLMs have great utility in assisting non-native English speakers with academic writing, representing a cost-effective and always-available alternative to commercial editing and proofreading services or to searching for native English-speaking collaborators (69).

Among the competencies listed in the ACGME’s Common Program Requirements is the ability to “systematically analyze practice using quality improvement (QI) methods” (20). GME trainees are required to participate in QI projects, which are typically require quantitative data analysis. Trainees are often underrepresented in organizational quality improvement activities, with one potential reason being the substantial time and effort needed for data collection and analysis (70). LLMs have some ability to facilitate straightforward data analysis and can generate serviceable code for statistical and programming tasks (71). LLMs are also adept at natural language processing tasks like extracting structured data from unstructured medical text (72).

### 2.5 Clinical decision support

Computer-based clinical decision support (CDS) systems are among the most effective tools for guiding good clinical decision-making (73). For GME trainees, CDS that provides authoritative, evidence-based guidance has both great practical clinical and educational utility (73). CDS that delivers evidence-based clinical guidance based on relevant patient data is a required feature for EHR systems certified by the United States government (74). A widely accepted CDS framework explains that CDS should be delivered according to the “five rights”: the right information, to the right person, in the right format, through the right channel, at the right time (75). Most current CDS consists of rule-based expert systems that display alerts to providers. While such systems are effective, rule-based alerts often suffer from practical problems such as a lack of specificity, poor timing, and incomplete characterization of clinical context (76).

The potential for intelligent, interactive, authoritative, LLMs to serve as always-available clinical consultants and educators has generated compelling speculation (77). LLMs can provide context-sensitive and specific guidance incorporating clinical context and patient data, they can be accessed through readily available communication channels, and--in contrast to rule-based alerts--they are interactive. However, studies done to evaluate the potential of LLMs for clinical decision support in various clinical contexts (78–83) have shown mixed results so far, with limitations in their ability to handle nuanced judgment and highly specialized decision-making. Thus, while GenAI for CDS is an area of great potential and ways to improve performance are under development, GME faculty and trainees cannot yet rely on LLMs to directly guide clinical care.

## 3 Risks

Despite its recent public availability, GenAI use is widespread and continues to grow quickly in both business and personal contexts. ChatGPT has the fastest-growing user base of any consumer web application in history (84), and a McKinsey & Company survey in early 2024 reported that 65% of businesses are regularly using generative AI, a rate nearly twice the year before (85). In another McKinsey report, more than 70% of healthcare leaders say they are using or pursuing GenAI technologies in their organizations (86). This explosive growth will undoubtedly have many benefits, but there are there are practical risks associated with GenAI that should temper optimism. Below we summarize the principal known risks as applicable in the GME setting:

### 3.1 Inaccuracy and overreliance

In essence, LLMs are statistical models that predict the most likely continuation of a given input sequence, based on their training data. Sometimes this approach results in plausible sounding but factually incorrect outputs, a phenomenon called “hallucination.” This problem can be especially difficult when dealing with topics requiring nuanced understanding of context or specialized knowledge, conditions very common in healthcare and specialized academic settings. For example, a biomedical researcher recently reported a cautionary tale in which ChatGPT generated incorrect information about tick ecology, complete with an entirely fake but plausible-appearing source document citation (87). In clinical settings, LLMs have been found to occasionally add fabricated information to clinical documentation (88) and to provide incorrect clinical recommendations (89).

In GME, trainees learn in a real clinical environment where accuracy and context are critical. There is a risk that overreliance on LLMs can result in an incomplete or incorrect understanding of complex topics, contributing to a poor educational outcomes, loss of critical thinking skills, and/or to suboptimal care and patient harm (15, 90, 91). Techniques like retrieval augmented generation, fine-tuning and prompt engineering show promise in reducing or eliminating the problems of inaccuracy and hallucination (92–94), but at present, reliance on GenAI as a source of factual information in any important clinical or academic context is risky. In our view, assertions made by GenAI should be validated by the user to avoid misinformation, and GME trainees should not use GenAI to directly guide patient care decisions outside of a controlled research context. GenAI users should be aware of automation bias, a cognitive bias in which people tend to excessively trust automated systems (95).

### 3.2 Authenticity and integrity

In reviewing applications for GME positions, personal statements are one of the most important elements that program directors review (96), especially in modern era where in-person residency and fellowship interviews are less common. Personal statements allow program directors to assess an applicant’s interest in their program and the clarity, organization and effectiveness of their written communication (97). There have long been concerns about plagiarism in personal statements (98, 99), and these concerns are magnified by GenAI tools that can readily produce writing that is clear, well-structured and compelling but that lacks an applicant’s unique voice, style and values (100, 101). Similarly, through letters of recommendation (LORs), faculty advocate for applicants by highlighting qualities observed in longitudinal relationships; using GenAI to draft LORs may have benefits but raises similar concerns about authenticity (102). GenAI-written content can be difficult to detect, even with software assistance (103). Some authors recommend that program draft policies for the use of GenAI in personal statements and LORs, with a common recommendation being that the use of GenAI be disclosed by the writer (97, 101).

As noted above, GME trainees are also expected to participate in research, academic writing, quality improvement summaries, creation of educational curricula, and similar academic activities. There are currently no consensus standards for using GenAI in academic medicine, but a recent review synthesized existing papers into a proposed set of guidelines, paraphrased as: (1) LLMs should not be cited as coauthors in academic works, (2) LLMs should not be used to produce the entirety of manuscript text, (3) authors should have an understanding of how LLMs work, (4) humans are accountable for content created by the LLM, and (5) use of an LLM should be clearly acknowledged in any resulting manuscripts (104).

### 3.3 Bias

GenAI tools are typically trained on huge corpora of data from the internet such as informational web sites, public forums, books, research literature, and other digitized media. Given the uncontrolled nature of the training data, it is unsurprising that they can exhibit social bias and stereotypes in their output (105). If unmanaged, these biases have the potential to reinforce detrimental beliefs and behaviors (106). In healthcare, GenAI may overrepresent, underrepresent or mis-characterize certain groups of people or certain medical conditions (18).

### 3.4 Privacy and security

GenAI is computationally intensive and expensive to operate. Thus, many resource-limited healthcare organizations or individual physicians may rely on third-party, external GenAI tools (107). Given the great utility of GenAI, knowledge workers may be sorely tempted to upload confidential information, despite significant risks (108). In healthcare, such risks are legal as well as ethical in nature, and transgressions can have implications for professional development.

## 4 Conclusion

Though the timeline is uncertain, GenAI technology will continue to advance. There is little question that GenAI will come to have a key role in the medical education landscape. We are optimistic about the potential of GenAI to enhance GME for both learners and educators, but enthusiasm should be tempered by a realistic understanding of the risks and limitations of this technology. We believe specific education on artificial intelligence should be included in medical curricula, and that research should continue on the risks and benefits of artificial intelligence as a tool for medical education (109, 110).
